# Responsibility loadings for dental services by general dentists

**DOI:** 10.1186/1472-6963-10-177

**Published:** 2010-06-23

**Authors:** David S Brennan, A John Spencer

**Affiliations:** 1 Australian Research Centre for Population Oral Health School of Dentistry, University of Adelaide, 5005, South Australia

## Abstract

**Background:**

Responsibility loadings determine relative value units of dental services that translate services into a common scale of work effort. The aims of this paper were to elicit responsibility loadings for a subset of dental services and to relate responsibility loadings to ratings of importance of the components of responsibility.

**Methods:**

Responsibility loadings and ratings of components of responsibility were collected using mailed questionnaires from a random sample of Australian private general practice dentists in 2007 (response rate = 77%).

**Results:**

Median responsibility loadings were 1.25 for an initial oral examination and for a 3+-surface amalgam restoration, 1.50 for a simple extraction and for root canal obturation (single canal), and 1.75 for subgingival curettage (per quadrant). Across the five services coefficients from a multivariate logit model showed that ratings of importance of knowledge (0.34), dexterity (0.24), physical effort (0.28) and mental effort (0.48) were associated with responsibility loadings (P < 0.05).

**Conclusions:**

The elicited median responsibility loadings showed agreement with previous estimates indicating convergent validity. Components of responsibility were associated with loadings indicating that components can explain and predict responsibility aspects of dental service provision.

## Background

In order to compare services either cross-sectionally or to examine trends over time a simple count of service items, while easy to interpret, suffers from the weakness of lack of comparability. For example, would a shift from restorative to diagnostic and preventive services equate to the same level of work effort? Relative value units (RVUs) provide a standardised unit of work effort for different types of service [1].

While a comprehensive set of estimates of RVUs exists in the literature [2], this set needs updating to accommodate new services and technology. Not only is there a need for a comprehensive set of estimates that can be applied to the full range of service items, there is also a need for greater transparency in understanding what is used to determine the estimates of relative value. The approach to estimating RVUs comprised multiplying a responsibility loading for each service by an estimate of time taken to perform the service. Responsibility loadings are typically related to factors such as knowledge, skill, and clinical and technical risk connected with a service [2]. Both time and know-how have been suggested as important factors in determining values for dental services [3]. However, there is little quantitative evidence of the factors that determine the responsibility loadings underlying the calculation of the relative value unit.

Validating, explaining and predicting responsibility loadings require further research. The aims of this study were to elicit responsibility loadings for a subset of dental services and to relate responsibility loadings to ratings of importance of the components of responsibility from a representative group of valuers (i.e., dental practitioners).

## Methods

### Sampling and data collection

Dentists were initially sampled at random in 1997 from the dental registers of each Australian State/Territory based on a sampling rate of 13.5%, resulting in a total sample of 1202 dentists. All dentists were eligible for sampling as specialists and those working solely in the public sector could not be identified from the sample frame, but were excluded later. Further details of sampling have been published previously [4-8]. In 2007 a total of 303 dentists from the pool of those 345 dentists who had provided data in the earlier study and were still registered were identified as still registered and included in the present study.

Data on responsibility loadings were elicited and ratings of components of responsibility were collected by mailed self-complete questionnaires [9]. A primary approach letter was sent to introduce the study, followed by the questionnaire a week later and up to four follow-up mailings were sent to non-respondents at intervals of approximately four weeks.

Analyses were restricted to private general practice dentists, as general practice comprised the highest main area of dental practice in Australia (85.1%), and the majority of dentists were from the private sector (82.6%) [10].

The research was approved by the Human Research Ethics Committee of the University of Adelaide.

### Data items

A set of 5 service items (see Table 1) was used to elicit responsibility loadings and to collect ratings of the importance of components of responsibility on 5-point Likert scales. These items were chosen to represent a range of values from previously published responsibility loadings drawn from a range of service areas (see Table 1 for a description of the items). Note that item 311 would be classified as an 'extraction' rather than a 'surgical extraction'. For each of the service items the previously published loading was provided and dentists were asked to choose their estimate of the loading (response categories provided were: 1.00, 1.25, 1.50, 1.75 plus an open-ended 'other:' please specify category). For each service item dentists rated the importance of knowledge, judgement, experience, dexterity, perception, physical effort and mental effort in relation to the responsibility loading for that service. Components of responsibility and intervention level have been based on previous reports from the dental [2,11], and medical literature [12]. Hypothesised components of responsibility include: Knowledge: grasp of facts or concepts relating to treatment (eg, basic sciences, materials, etc), Judgement: ability to make decisions based on evidence (eg, clinical knowledge, case presentation), Experience: familiarity with the service (eg, frequency and recency of performing the task), Dexterity: digital dexterity (eg, motor skills, co-ordination) required for the execution of the task, Perception: perceptual ability (eg, visual, tactile skills) required for the execution of the task, Physical effort: the physical effort (eg, strength, endurance) required to perform the task, and Mental effort: the mental or cognitive effort (eg, concentration) required to perform the task.

**Table 1:** Descriptive statistics of responsibility (R) loadings for dental services

			R loading statistics from this study					
			
						Percentile		
						
Item Code	Description of service item	^(a)^Published R loading	n	Mean	SD	25%	Median	75%
011	Initial oral examination	1.25	194	1.41	0.24	1.25	1.25	1.50
513	Amalgam restoration - three or more surfaces - permanent tooth	1.25	190	1.35	0.22	1.25	1.25	1.50
311	Removal of a tooth or part(s) thereof	1.50	187	1.53	0.23	1.50	1.50	1.75
417	Root canal obturation - one canal	1.50	193	1.57	0.19	1.50	1.50	1.75
211	Subgingival curettage, per quadrant	1.75	187	1.63	0.23	1.50	1.75	1.75

(a): Clappison RA, et al. *J Can Dent Assoc *1965; 31: 763-778.

### Data analysis

The age and sex profile of respondents to the earlier study were compared against published estimates of the dentist population. Response rate to the present study and bias were investigated by comparing the characteristics of dentists who provided data in the present study against those dentists who provided data in the earlier study only.

Descriptive statistics for the responsibility loadings were determined. Both means and medians are reported along with standard deviations and 25^th ^and 75^th ^percentiles to allow comparison of point estimates and assessment of variability around these estimates. The five-point Likert scales ranging from 1 = low importance to 5 = high importance for each of the components of responsibility were recoded into dichotomous variables where the values 4 and 5 where aggregated to represent a high rating of importance for an item, else the values 1 to 3 were aggregated to represent a lower rating of importance for an item. The percentage rating a service item as important was tabulated for each item to assess the association of each component with each service item. Multivariate ordered logit models were estimated using the responsibility loading as the dependent variable with indicator variables for each component of responsibility as the independent variables (coded 1 = high importance, 0 = lower importance). While some loss of sensitivity may be associated with the dichotomous coding of the independent variables the meaning of the recoded variables may be more readily interpreted in terms of importance. An ordered logit model was fit as responsibility loadings tend to have a small range of values that fall into ordered categories. The combined logit model for all five services was adjusted for the clustering effect of multiple observations (i.e., five services combined) per dentist [13].

## Results

### Response

A total of 676 dentists responded to the earlier study in 1997-98, resulting in a response rate of 60.3%. The percentage of female dentists (20.0%) was close to the dentist population (23%), and the responding practitioners also had an age distribution that was similar to the dentist population [13].

In the present study in 2007 a total of 215 dentists responded (response rate = 77.3%, adjusted for those who were out of scope because they were no longer at their sampled address). Compared to the dentists who responded to the earlier study, but not to the present study, the characteristics of the respondents were similar in terms of sex, geographic location (capital city, non-capital), practice type (solo, partnership, associateship, assistant, other), percent of time spent in their main practice, numbers of patients treated (per hour, per year, per day), time worked (hours per year), waiting time for an appointment, and numbers of staff (numbers of other dentists, assistants, hygienists, managers, secretaries, and other types of personnel). The only observed difference was for age of dentist, which was older in 1997 for dentists who responded to the earlier study only.

### Responsibility loadings

The mean elicited responsibility loadings ranged from 1.35 for an amalgam restoration on a permanent tooth (three or more surfaces) to 1.63 for subgingival curettage (Table 1). The percentiles indicated that while there was some spread in the data, the median for each item was identical to previous published estimates.

### Components of responsibility

The percentage of dentists rating each component as important in determining responsibility loadings for each service is presented in Table 2. Overall, knowledge, judgement and experience were rated most highly. This reflected the response for an oral examination and root canal obturation. For subgingival curettage experience and dexterity were most highly rated, followed by knowledge and judgement. For removal of a tooth, experience was most highly rated, followed by knowledge, judgement and dexterity. For a restoration, dexterity and experience were most highly rated.

**Table 2:** Percentage of dentists rating component as important in determining responsibility loading for a service

	Item code					
		
Component	011 Initial oral exam	513 Amalgam filling	311 Tooth removal	417 Root canal treatment	211 Subgingival curettage	All
Knowledge	87.3	44.9	72.6	88.3	75.0	73.7
Judgement	90.4	50.8	77.6	81.7	71.9	74.5
Experience	81.0	61.3	84.3	85.8	82.7	79.1
Dexterity	12.1	73.9	72.6	78.2	86.2	64.5
Perception	60.6	39.4	53.8	67.7	69.2	58.2
Physical effort	5.6	32.8	71.6	33.7	65.1	41.7
Mental effort	58.2	32.5	57.1	59.0	52.8	52.0
						
All	56.4	47.9	69.9	70.7	71.9	63.4

### Associations of components of responsibility with loadings

The multivariate models provide estimates of strength and direction of associations of components with responsibility loading that are adjusted for the other components in the model (Table 3). For all items combined, knowledge, dexterity, physical effort and mental effort were significantly associated with responsibility loadings across the five services. Knowledge was significantly associated with responsibility loadings for provision of a restoration and removal of a tooth. Mental effort was significantly associated with provision of an oral examination, a restoration, removal of a tooth, and curettage.

**Table 3:** Coefficients (SE) from ordered logit models of responsibility loading for a service by components

	Item code											
		
Component	011 Initial oral exam		513 Amalgam filling		311 Tooth removal		417 Root canal treatment		211 Subgingival curettage		All	
	**Coeff**.	**(SE)**	**Coeff**.	**(SE)**	**Coeff**.	**(SE)**	**Coeff**.	**(SE)**	**Coeff**.	**(SE)**	**Coeff**.	**(SE)**
	
Knowledge	0.05	(.22)	*0.64	(.27)	*0.49	(.25)	0.52	(.31)	0.24	(.24)	*0.39	(.11)
Judgement	0.23	(.27)	0.14	(.27)	0.35	(.28)	0.01	(.26)	-0.08	(.26)	0.11	(.12)
Experience	0.19	(.19)	0.02	(.22)	0.17	(.25)	-0.35	(.27)	0.44	(.28)	0.06	(.11)
Dexterity	-0.11	(.20)	0.09	(.25)	0.15	(.22)	0.37	(.25)	0.15	(.29)	*0.24	(.08)
Perception	0.06	(.15)	-0.40	(.21)	-0.17	(.21)	0.11	(.21)	0.31	(.24)	-0.01	(.10)
Physical effort	0.11	(.21)	0.29	(.21)	0.10	(.20)	0.17	(.16)	0.23	(.19)	*0.28	(.08)
Mental effort	*0.68	(.17)	*0.55	(.23)	*0.52	(.20)	0.35	(.20)	*0.44	(.22)	*0.48	(.11)
												
**Model P-value**	**		**		**		**		**		**	
**Pseudo R-sq**.	**7%**		**12%**		**13%**		**8%**		**11%**		**11%**	

*P < 0.05

**P < 0.01

## Discussion

The study showed that responsibility loadings elicited from a sample of dentists were similar to those from previous reports based on expert panels demonstrating convergent validity over time and method of elicitation. The components of responsibility tended to vary by service item and responsibility, providing explanation of what comprises the responsibility dimension of dental service delivery and suggesting the utility of using components to predict responsibility loadings.

### Relative value units

The comparison of services inherent in the RVU approach tends to use a defined base service such as an occlusal amalgam restoration which is given a responsibility loading of 1.0 and other services can be valued in relation to this reference point. Additional factors are required to convert RVUs into monetary values taking into account factors such as laboratory costs, office overhead, and cost of materials [2].

However, a range of approaches to establishing a standardised unit of work exists and there is a lack of consensus as to the optimal method. Swedish studies have used standardised units, the Dental Service Unit, based on direct time studies of dental procedures [14]. Studies in the USA have calculated relative values for items to assist dentists in setting fees [10]. British studies have shown differences between resource-related indices used in epidemiological studies [15]. Weights of dental services used in the relative value unit approach reflect in part the result of subjective or negotiated judgements, which may vary over time.

Other approaches to valuing dental procedures include relative time-cost units, based on personnel costs, task mixes, and task times involved in dental procedures [16]. These approaches suffer from problems of subjectivity and lack of a comprehensive set of units that can be applied to the full range of dental services provided. Studies based on time units [14,15], are limited by the restriction to time as the only measure of relative value of services, without accounting for other factors such as responsibility associated with service provision. The relative values produced by the Council on Dental Health [10], comprised a set of values for a large set of treatment items but details of the component dimensions of each item were not listed hindering the transparency of the valuing process, the values were derived from only two panels of 25 dentists, perhaps limiting the representativeness of the estimates.

### Limitations

The response at both points in time was adequate (i.e., 60% and 77%). Previous reports of responses of Australian dentists to mailed surveys have shown response across mailing stages of approximately 20%, 20%, 10%, 5% and 5% respectively [17], indicating both diminishing returns across stages and the necessity for multiple follow-ups to achieve acceptable response levels. There was little evidence of response bias, except for the difference in age of dentist between those who responded to the present study, and those who responded to the earlier study only. However, this is consistent with the loss to follow-up of older dentists who responded earlier, but subsequently were no longer registered at the time of the present study which occurred 10 years later.

It has previously been noted that dentists who perform a type of service less frequently may consider such a service to be more complex and demanding than dentists who perform the service more frequently [3]. Hence valuation of a service may at least partly reflect the frequency and familiarity of a service. It is possible that specialists may rate services differently from general practice dentists. Hence the findings presented are restricted to private general practice dentists. This phenomenon is consistent with service rate distributions as the services with lower responsibility loadings (i.e., initial oral exam and amalgam restoration) are from main areas of service that are provided at higher rates (i.e., diagnostic and restorative services) [4]. However, it should be noted that while the responsibility loadings for an oral exam and amalgam restoration were similar the components comprising the loadings varied. For example, an initial exam had higher ratings for the components of knowledge, judgement and perception but lower ratings for components such as dexterity and physical effort compared to an amalgam restoration.

This study used a selected subset of service items to assess the utility of the approach. Further research is needed to expand the service items and to examine the potential to predict responsibility loadings from estimates of components of their importance.

Relative value studies tend to provide reference values at least as anchor points for valuation of other services. For example, a two-surface amalgam filling has been used as a reference procedure against which to value other procedures [3]. In this study previously published responsibility loadings were disclosed to study participants so it is possible that respondents could be influenced to some extent in their choice of values by knowledge of previous values. While level of agreement with published values of responsibility loadings were exact at the median level, the mean values and the measures of dispersion show that there was some variability in the stated values between dentists.

### Significance

RVUs have application in the area of health financing as a payment mechanism as well as a practice management tool [18]. RVUs can be used as an objective measure of productivity, such as comparisons based on RVUs per hour [19]. RVUs can also be applied to cost analysis and to benchmarking [20,21]. The information about responsibility loadings and their components could also be useful in informing the public about the rational basis of setting fees.

## Conclusions

These findings demonstrate agreement in elicited median responsibility loadings with previous estimates indicating convergent validity. Components of responsibility were associated with responsibility loadings indicating that components such as knowledge, dexterity and effort can be used to explain and predict responsibility aspects of dental service provision.

## Competing interests

The authors declare that they have no competing interests.

## Authors' contributions

DSB performed data analysis and writing of the manuscript. AJS provided advice and contributed to finalising the manuscript. DSB and AJS were chief investigators on the ADRF grant. Both authors read and approved the manuscript.

## Pre-publication history

The pre-publication history for this paper can be accessed here:

http://www.biomedcentral.com/1472-6963/10/177/prepub
